# Validating the Impact of Water Potential and Temperature on Seed Germination of Wheat (*Triticum aestivum* L.) via Hydrothermal Time Model

**DOI:** 10.3390/life12070983

**Published:** 2022-06-30

**Authors:** Saleha Saeed, Abd Ullah, Sami Ullah, Javaria Noor, Baber Ali, Muhammad Nauman Khan, Mohamed Hashem, Yasser S. Mostafa, Saad Alamri

**Affiliations:** 1 Department of Botany, University of Peshawar, Peshawar 25120, Pakistan; salehasaeed36@gmail.com; 2 Xinjiang Key Desert Plant Roots Ecology and Vegetation Restoration Laboratory, Xinjiang Institute of Ecology and Geography, Chinese Academy of Sciences, Urumqi 830011, China; 3 State Key Laboratory of Desert and Oasis Ecology, Xinjiang Institute of Ecology and Geography, Chinese Academy of Sciences, Urumqi 830011, China; 4 University of Chinese Academy of Sciences, Beijing 100049, China; 5 Department of Botany, Islamia College Peshawar, Peshawar 25120, Pakistan; jnoorbotanist@gmail.com (J.N.); nomiflora@uop.edu.pk (M.N.K.); 6 Department of Plant Sciences, Quaid-i-Azam University, Islamabad 45320, Pakistan; baberali@bs.qau.edu.pk; 7 Department of Biology, College of Science, King Khalid University, Abha 61413, Saudi Arabia; mhashem@kku.edu.sa (M.H.); Ysmosutafa@kku.edu.sa (Y.S.M.); saralomari@kku.edu.sa (S.A.); 8 Faculty of Science, Botany and Microbiology Department, Assiut University, Assiut 71516, Egypt

**Keywords:** water potential, cardinal temperatures, hydrotime model, hydrothermal time, wheat

## Abstract

Wheat is the most extensively cultivated crop and occupies a central place in human nutrition providing 20% of the daily food calories. This study was conducted to find both T and ψ effects on wheat germination and the cardinal Ts value; a lab experiment was accomplished using HTT models. Cultivars were germinated under different accelerated aging periods (AAP, 0, 24, 48, and 72 h) at each of the following constant Ts of 15, 20, 25, 30, and 35 °C at each of the ψs of 0, −0.05, −0.1, −0.15, and −0.2 MPa. GR, GP, and other germination parameters (GI, GRI, CVG, SVI-I, SVI-II, GE, and MGT) were significantly determined by solute potential, temperature, and reciprocal action in both cultivars (*p* ≤ 0.01). Depending on the confidence interval of the model co-efficiently between cultivars, there was no significant difference. Hence, the average of cardinal Ts was 15, 20, and 35 °C for the Tb, To, and Tc, respectively, in the control condition (0 MPa). Hydro-time values declined when Ts was raised to To in cultivars, then remained constant at Ts ≥ To (2.4 MPah^−1^ in Pirsabak 15 and 0.96 MPah^−1^ in Shahkar). The slope of the relationship between ψ_b(50)_ and TTsupra with temperature when Ts is raised above To and reaches 0 at Tc. In conclusion, the assessed parameter values in this study can easily be used in simulation models of wheat germination to quantitatively characterize the physiological status of wheat seed populations at different Ts and ψs.

## 1. Introduction

Cereals, such as wheat, maize, and rice, are among the most important sources of calories and protein worldwide. Wheat was the first domesticated crop and is now the most important staple crop worldwide [1]. Based on estimates, wheat accounts for 38.8% of the harvested area and provides significantly more protein per gram (12–15%) compared to rice or maize (2–3%), thus serving as a more advantageous cereal [2]. Despite the fact that it is cultivated on a large area of land, its production levels are much lower than those of maize and rice [3]. A meta-analysis of 17,006 simulations shows that every 2 °C increase in temperature greatly reduces its productivity in temperate and tropical regions [4]. In a similar climate study, the researchers predicted that wheat yields would reduce by 6%, equivalent to a possible 42 Mt/°C [5]. The continuous change in climate conditions has led to an increase in environmental stresses, which negatively impacts the development, growth, and productivity of important crop species, including wheat [6,7,8,9]. Therefore, adaptation strategies need to be designed to maximize yield for continuously increasing food demands in the face of continuously changing climatic conditions.

Seed germination (SG) is a critical phase of plant development primarily affected by abiotic stressors [10,11,12,13]. Temperature and water potential have been determined to be major determinants of germination rate (GR), while other factors were not relevant for non-dormant seeds (Bradford 2002). In order to understand how and why germination in various environmental conditions is affected, seedling growth models are employed. A number of studies have examined the effects of temperature (T), water potential (ψ), and the interaction between T × ψ on germinating seeds via thermal, hydrothermal, and hydrotime models [7,13,14].

Temperature (T) and water potential (ψ) are the two critical environmental factors affecting seed germination rates, germination percentages, seedling emergence and establishment [15]. It is fairly helpful to use different models for predicting the response of seed germination and emergence of seedlings to various abiotic stress factors. For instance, several studies have utilized the hydrothermal model (HTT) and the hydrotime model (HT) to analyze the responses of seed germination to changes in temperature and water [16,17,18,19].

In addition, to determine the best planting date for each crop, it is important to understand the three fundamental concepts of solar gradient: base (Tb, SG = zero), optimum (To, SG = maximum), and ceiling temperature (Tc, SG = zero) [18,20,21]. Furthermore, the accelerated aging test is a straightforward, quick, and low-cost method of ranking seeds based on seed degradability and vigor. When the accelerated aging period (AAP) increases, the characteristics of SG decreases in different crop plants, including wheat [22,23].

The hydrothermal (HTT) time model measures the germination time concept across T and ψ in the sub-optimal range (between Tb-To) and with alteration, as well as in the supra-optimal range (between Tb-Tc [7]. So far, several species have adopted this approach. For instance, *Daucus carota* [24], *Plantago ovata* Forssk. [25], *Sinapis arvensis* L. [26], *Eruca sativa* [7], and *Hordeum vulgare* [13]. To our knowledge, research regarding hydrothermal time validation for predicting wheat germination to different water potential and temperature levels is scarce. Hence, we aimed to investigate the effect of both T and ψ on seed germination and the cardinal Ts value of wheat using HTT models.

In the present study, we aimed (1) to predict the response of wheat germination at various temperatures and solute potentials; (2) to influence the Ψ_b_ and the cardinal temperature for this plant; (3) to determine the effects of accelerating the aging period on the response of wheat germination under different solute potentials using the hydrotime concept.

## 2. Materials and Methods

### 2.1. Seed Description and Experimental Protocol

Two winter varieties of wheat seeds, including “Pirsabak 15” and “Shahkar”, were obtained from the cereal crop research institute (CCRI) Pirsabak, Nowshera. Seeds were assembled at the University of Peshawar, Pakistan, in November 2020. The main plot consisted of four levels of the accelerated aging period (AAP; 0, 24, 48, and 72 h), with five levels of water potential (0, −0.05, −0.15, and −0.2 MPa) and a range of constant Ts (15, 20, 25, 30, and 35 °C) by using incubator (Memmert Beschickung-Loading Model 100–800). As a control, distilled water was used (0 MPa), and the solute potential was formed using a liquid solution of PEG6000, followed by Michel and Radcliffe (1995). According to [7], seeds were germinated in 95 mm Petri dishes. Concisely, 10 seeds were kept in each Petri dish, with 2 layers of Whatman No. 1 filter paper and control, 5 mL of distilled water, or 5 mL of polyethylene glycol solution. At each temperature, 3 replicates were generated for the respective treatment. Petri dishes were randomly incubated inside the dark (incubator), excluding when noted SG. Depending on the T and Ψ, seeds were reported frequently daily, and when the radical was at least 0.2 cm long, the seeds were considered to have germinated. To avoid errors, we removed germinated seeds when recording germination in each Petri dish. When we had no new germinated seeds at each cardinal T, the experiment was finished in each treatment replicate for five successive days. The hydrothermal time model was fitted using the statistical analysis system IBM SPSS Statistics 2020 and Excel software, and figures were drawn by the origin 2020 software. According to the method reported in [17,20], the hydrothermal time model parameters were influenced.

### 2.2. Data Analysis

The germination data were evaluated using a repeated probit regression analysis based on the TT, HT, and HTT models, as stated by [7,25]. For each percentile at each T or C, the germination rate (GR) was computed as the inverse of the germination time.

### 2.3. Thermal Time (TT)

For deciding the appropriate water level and temperature for effective crop development, three cardinal temperatures (Ts) include maximum temperature (Tc), base temperature (Tb), and optimum temperature (To) [7,21,25]. The thermal time model can be arranged as:

(1) TTsub=(T−Tb)tg at sub−optimal T

(2) TTsupra=(Tc−T)tg at supra−optimal T

### 2.4. Hydrotime (θH)

Gummerson (1986) suggested using a hydrotime model (θH) to improve the model prediction. θH determines the relationships between solute potential and germination rate in the same way as the thermal time model:

(3) $$\theta H\left(g\right)=\left(\psi -\psi_{b}\right)\mathrm{tg}$$

(4) $$\mathrm{probit}\;g=\left\{\;\psi -\left(\theta H/\mathrm{tg}\right)-\psi_{b\;50}\right\}/\sigma \psi_{b}$$

### 2.5. Hydrothermal Time Model (HTT)

The hydrothermal time model (HTT) was a combination of the thermal time model (TT) and the hydrotime model (θH) [16].

(5) $$\theta \mathrm{HTT}=\left(\psi -\psi_{b(g)}\right)\;\left(T\;-\mathrm{Tb}\right)\;\mathrm{tg}$$

(6) $$\mathrm{probit}\;g=[\left(\psi \;\left(\theta \mathrm{HTT}/\left(T-\mathrm{Tb}\right)\mathrm{tg}\right)-\psi_{b\;(50)}\;\right]/\sigma \psi_{b}$$

Within the seed lot, population σψ_b_ is the standard deviation of Ψ_b_, and Ψ_b(50)_ is the base water potential of the 50th percentile. The amount of Ψ_b(g)_ varied among seeds in the population, and the θHTT and base temperature are expected to be constant in this model [16]. Nevertheless, the models could not claim a decrease in germination rate at Ts [17]. Previous studies on potato (*Solanum tuberosum* L.) [17], lemon balm (*Melissa officinalis* L.) [18], watermelon (*Citrullus vulgaris* L.) [7], and zucchini (*Cucurbita pepo* L.) [27] reported that there were interactions between temperature and water potential at a supra-optimal range of T. In all these publications, there is an increase in the amount of Ψ_b(g)_ as temperature rises above the optimum temperature. Nevertheless, a modified form of Equation (5) was found [28].

(7) $$\theta \mathrm{HTT}=\left[\psi \;-\psi_{b(g)}\;-\;\left(k_{T}\;\left(T\;-\;\mathrm{To}\right)\right)\right]\;\left(T\;-\;\mathrm{Tb}\right)\;\mathrm{tg}$$

(8) $$\mathrm{probit}\;g=\left[\psi \;-\;k_{T}\;\left(T\;-\;\mathrm{To}\right)-\left(\theta H/\left(T-\mathrm{Tb}\right)\;\mathrm{tg}\right)-\psi_{b\;(50)}\;\right]/\sigma \psi_{b}$$

where k_T_ is a constant (the slope of the relationship between Ψ_b(50)_ and Ts > To, Alvarado and Bradford (2002) reported that the value of Ψ_b(50)_ is equal to Ψ_b(50)_ distribution at To and T-To is equal to To-Tb at the supra optimal range of Ts. The shift in the distribution of ψ_b(g)_ with T is linear at Ts > To the models (Equations (7) and (8)), which could be used. Studies by [27,29] described methods for the TT sub-optimal, TT supra-optimal, hydrotime, and hydrothermal time models (Equations (1)–(8). Equations (5) and (7) combine the HTT model and are used to predict and describe seed germination responses at all cardinal temperatures and water potentials at which germination could occur.

### 2.6. Germination Attributes

The per day and cumulative germination, physical observation, radicle, and plumule lengths, and fresh and dried weight of the seedlings were used to generate the following germination indices.

#### 2.6.1. Mean Germination Time (MGT)

MGT is a measure of how rapidly a population of seeds germinated. The higher the population germinated, the lower the mean germination time [30].

(9) $$\mathrm{MGT}=\frac{\sum \mathrm{fx}}{\sum f}$$

The number of seeds germinated on day x is denoted by the letter “f”.

#### 2.6.2. Germination Rate Index (GRI)

Higher GRI values indicate more excellent and maximum GR. GRI represents the percentage of SG overtime throughout the germination phase [30].

(10) $$\mathrm{Germination}\;\mathrm{Rate}\;\mathrm{Index}\;=\frac{G1}{2}+\frac{G2}{2}+\ldots \ldots +\frac{\mathrm{Gx}}{2}$$

G1 represents the percentage of germinated seeds on the first day after planting, whereas G2 represents the percentage of germinated seeds on the second day after sowing.

#### 2.6.3. Germination Index (GI)

The germination index was calculated using a standard procedure [30].

(11) GI =(10× n1)+(9× n2)+……+(1× n10)

Thus, n1, n2, …, n10 denoted the number of seeds germinated on the first, second, and third days, respectively, while 10, 9, …, and 1 denoted the number of germinated seeds on the first, second, and third days, respectively.

#### 2.6.4. Coefficient of Velocity of Germination (CVG)

The coefficient of germination reflects the speed with which seeds germinate. The lower the time, the higher the CVG value necessary for germination. When all seeds germinate on the first day, the most outstanding CVG value (100) may be attainable [30].

(12) $$\mathrm{CVG}=N1\pm N2\pm \ldots \pm \frac{\mathrm{NX}*N1T1+\ldots \ldots N\times T}{100}$$

where “N” is the number of seeds germinate each day, and temperature denotes the number of days from planting corresponding to seed germinated N.

#### 2.6.5. Germination Energy (GE)

We determined germination energy following a standard technique [31].

(13) $$\mathrm{GE}=\frac{X1}{X2}+\frac{\left(X2-X1\right)}{Y2}\ldots \ldots +\frac{\left(\mathrm{Xn}-\mathrm{Xn}-1\right)}{\mathrm{Yn}}$$

The final germination on the last (nth) counting day is Xn, and the number of days from sowing to the previous (nth) counting date is Yn.

#### 2.6.6. Seed Vigor Index-I (SVI-I)

Seed vigor was calculated using the following equation.

(14) Seed vigor index=seedling length (cm)×seedling germination %age

#### 2.6.7. Seed Vigor Index-II (SVI-II)

Seed vigor index was determined using the following Equation.

(15) Seed vigor index=Seedling dry weight (mg)× Seed germination % age

### 2.7. Statistical Analysis

Using IBM SPSS Statistics 26 and SigmaPlot Version 11.0, the effects of Ts (thermal time) (hydrotime) and their interactions (hydrothermal time model) on seed germination rate and germination characteristics were investigated using linear regression. Excel was used to conduct the basic statistical calculations. The values of the following parameters were calculated using linear probit regression analysis in SPSS: ψ_b(50)_, ψ_b_, R^2^, SE. To produce various graphs of germination fraction vs. accelerated ageing duration and germination parameters versus T and C, the ORIGIN 2021 PC Corporation was utilized.

## 3. Results

**Figure 1:**
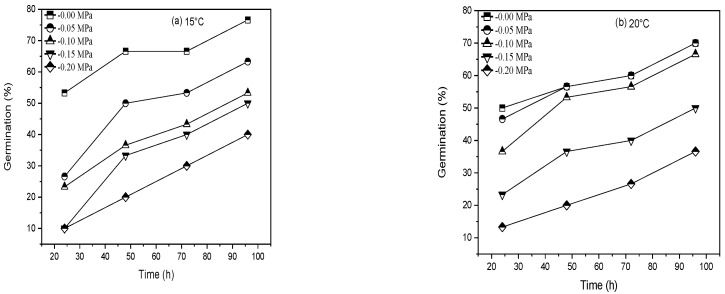

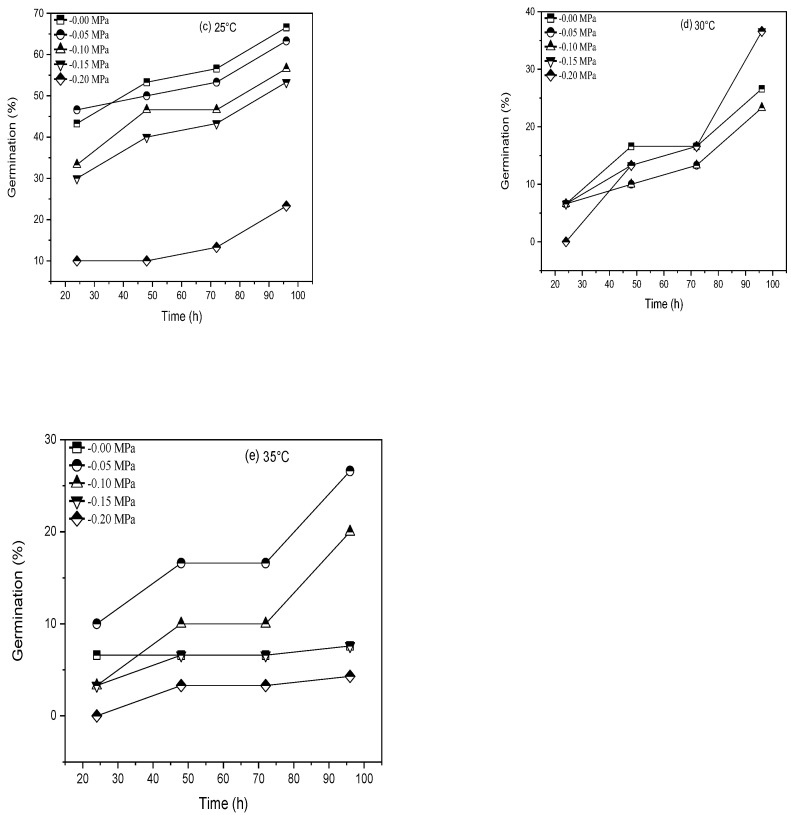
(**a**–**e**) Interaction effect of water potential and accelerated aging period on the germination percentage of *Triticum aestivum* L. Pirsabak 15 at 15, 20, 25.30, and 35 °C.

**Figure 2:**
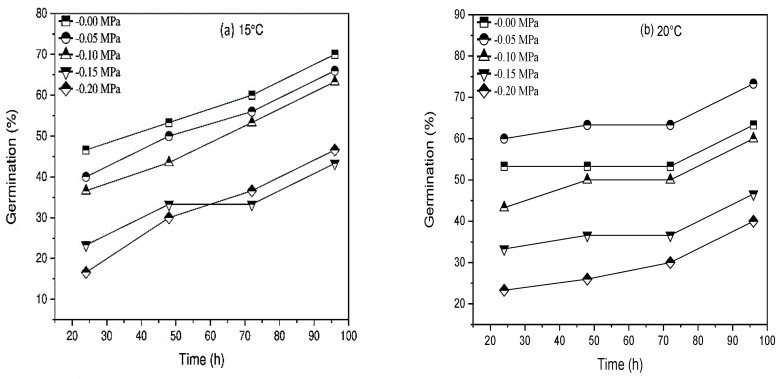

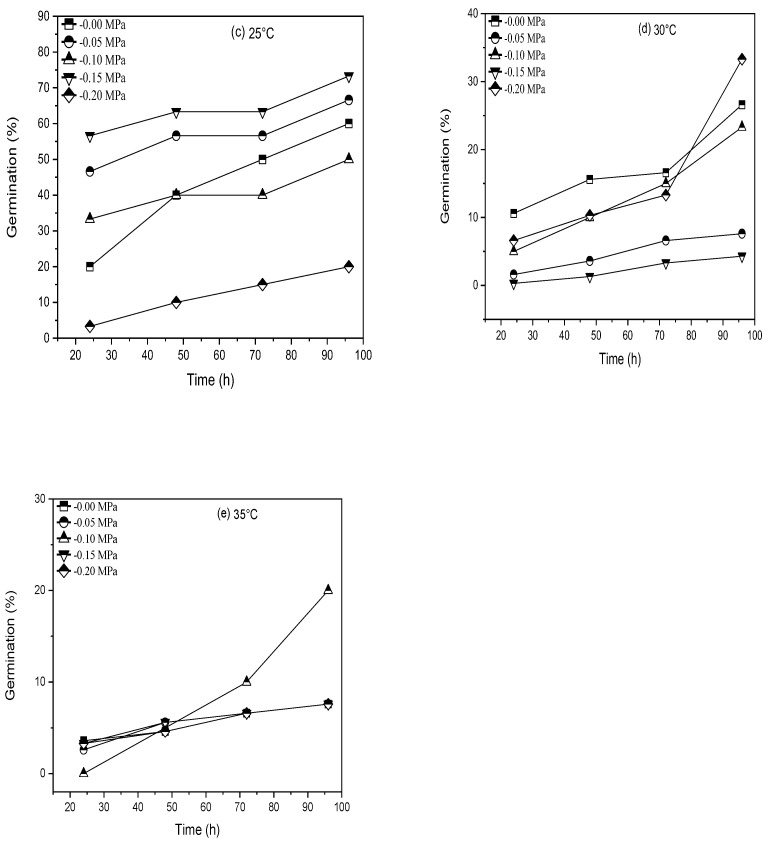
(**a**–**e**) Interaction effect of water potential and accelerated aging period on the germination percentage of *Triticum aestivum* L. Shahkar at 15, 20, 25, 30, and 35 °C.

**Figure 3:**
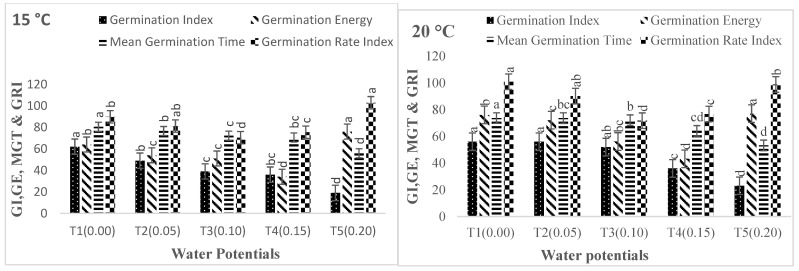

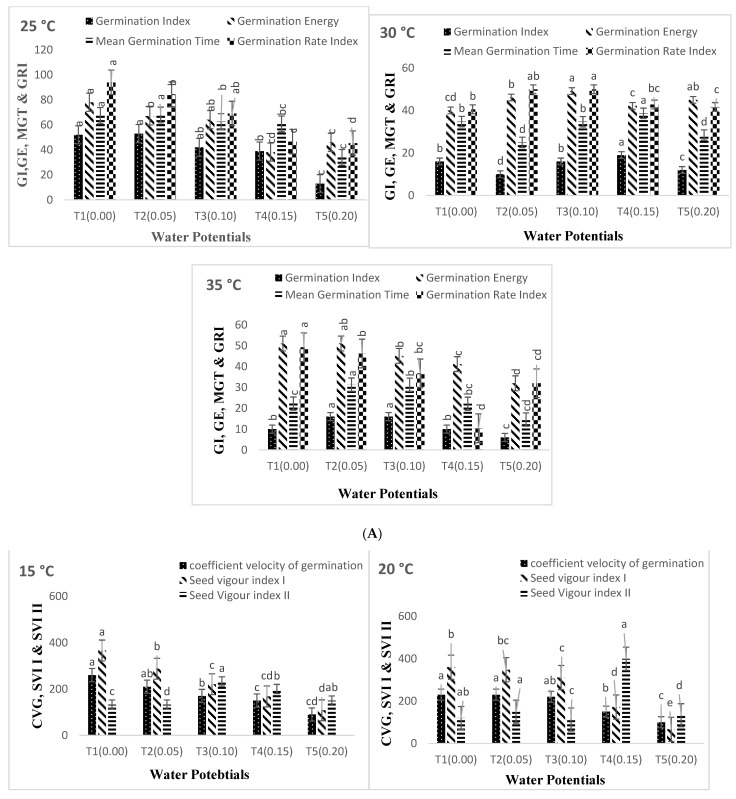

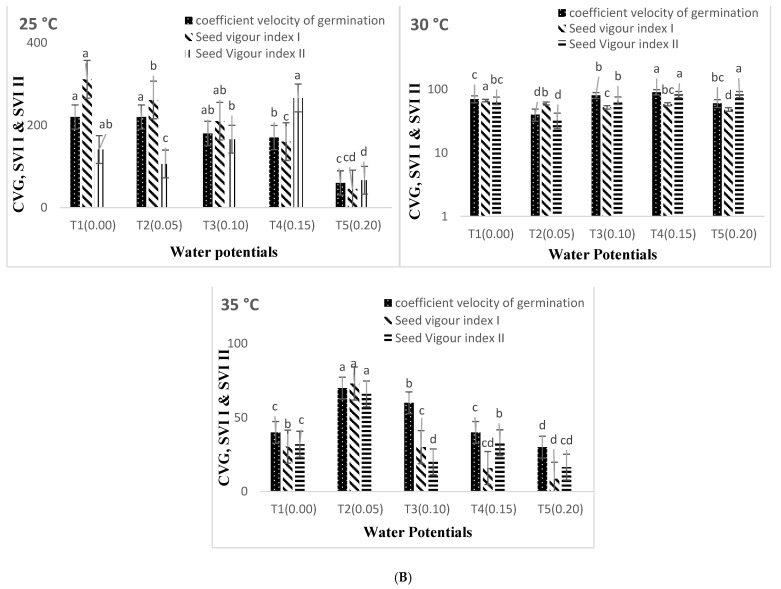
(**A**) Interaction effect of water potential and temperature on germination parameters (MGT, GI, GRI, GE) of *Triticum aestivum* L. Pirsabak 15 during the aging period (24, 48, 72, 96). (**B**) Interaction effect of water potential and Temperature on Germination parameters (CVG, SVI I, and SVI II) of *Triticum aestivum* L. Pirsabak 15 during the aging period (24, 48, 72, 96). Different letters above the columns represent significant differences at *p* < 0.05 (LSD method) method.

**Figure 4:**
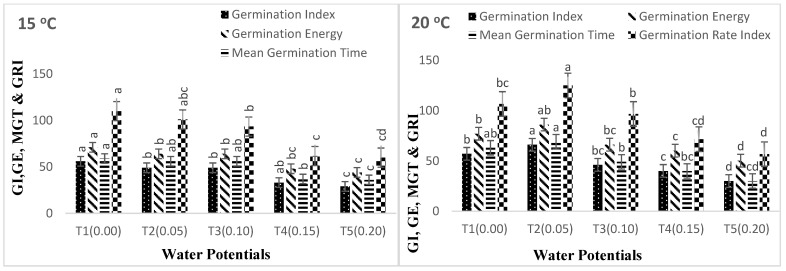

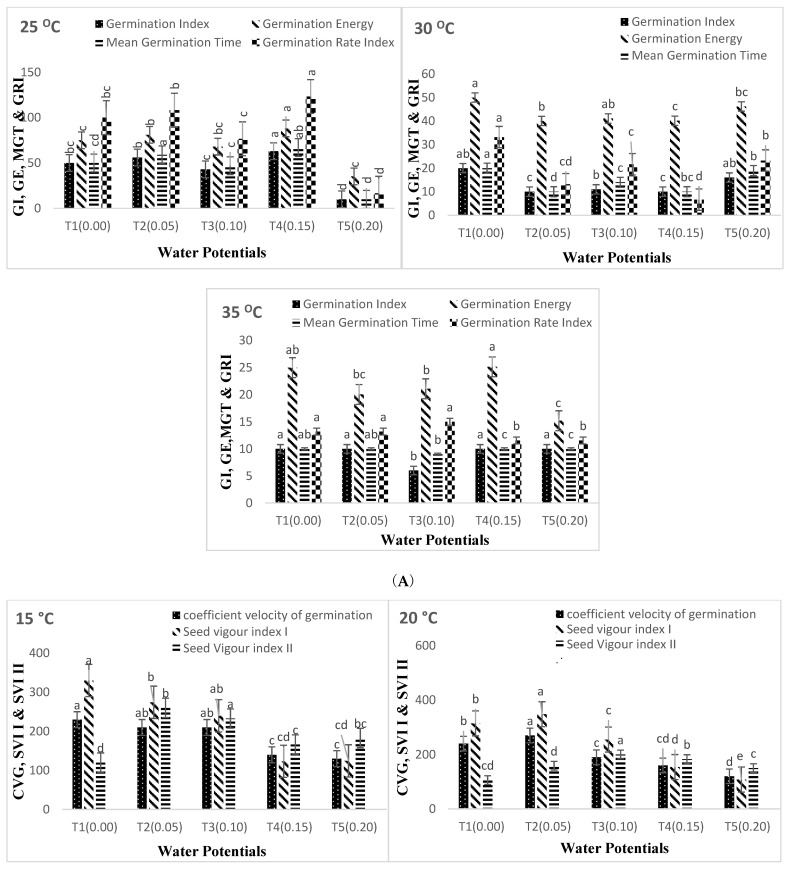

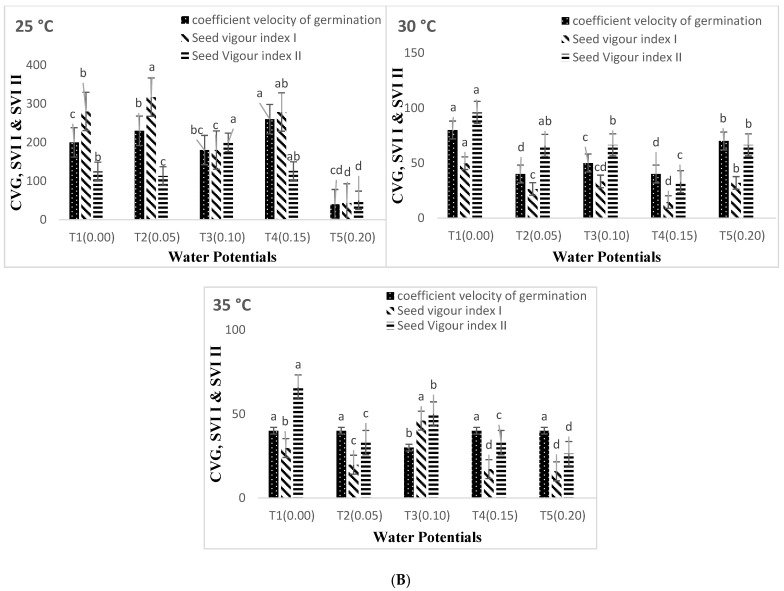
(**A**) Interaction effect of water potential and temperature on germination parameters (MGT, GI, GRI, GE) of *Triticum aestivum* L. Shankar 15 at aging period (24, 48, 72, 96). (**B**) Interaction effect of water potential and Temperature on germination parameters (CVG, SVI I and SVI II) of *Triticum aestivum* L. Shankar 15 at aging period (24, 48, 72, 96). Different letters above the columns represent significant differences at *p* < 0.05 (LSD method) method.

Our findings revealed that ψ, Ts, and AAP and their interactions substantially affected the germination percentage and other characteristics (Figure 1, Figure 2, Figure 3 and Figure 4). The longer and lower AAP caused obvious changes in seed percent germination (Figure 1 and Figure 2). Longer AAP (from 0 to 24, 48, and 72 h) significantly reduces GP compared to control (averaged across all levels). Furthermore, when GP was increased from zero to −0.05, −0.1, −0.15, and −0.20 MPa, it was reduced in contrast to the control (averaged for all levels of AAP) (Figure 1 and Figure 2).

Water potential, temperature, and inter-linkage (*p* ≤ 0.01) significantly affected the germination percentage and germination rate of both varieties of *Triticum aestivum*. Likewise, when Ψ declined at each T, the germination rate and percentage decreased. The θH was constant at 2.4 MPah^−1^ for Pirsabak 15 and 0.96 MPah^−1^ for Shahkar. The water relations changed when Ts increased above To but not at sub-optimal Ts of temperature (Table 1) when Ts decreased below To. Rather, the θH value was raised as the tempera model proposed by Alvarado and Bradford (2002) to report the relationship between Ψ_b(50)_ and temperature at supra-optimal Ts (Figure 1 and Figure 2). Take hold of the point of the model Ψs = 0 MPa at the Tc value, which was concluded from the germination rate data (Figure 1 and Figure 2) and or measured by the fitting of Equation (8) (Table 2), based on the confidence interval of the models. There was no significant difference between varieties, so the mean value of Tc and k_T_ was approximately 0.1041 MPaOCh^−1^ for both types (Table 2). On the contrary, this shows that for each °C raised at T > To, the effect on germination seed water potential was increased and became more positive by 0.1041 MPa.

Following the hydrothermal time model coefficient, *Triticum aestivum* germination was ventilated in water when temperature declined at sub-opt TT. The Ψ_b(50)_ value of *Triticum aestivum* varieties was varied with temperature, and it remained constant at Ts<to< span=""> (−0.12 MPa, averaged for both varieties, calculated by using Equation (8) and then raised linearly with temperature when Ts was raised above To (Figure 1 and Figure 2). Depending on the temperature in both varieties, the amount of σψ</To < ψ_b_ varied and ranged from 0.031 to 0.161 MPa. According to the outcomes, Ψ_b(50)_ values were raised from −0.12 MPa at 20 °C (To) to −0.015, −0.02 MPa for “Pirsabak 15” and “Shahkar” cultivars severally at 30 °C, when the temperature was raised above the optimum temperature (Table 1 and Figure 1). Consequently, the line temperature was equal to the maximum temperature or when the base water potential was 0 MPa (Figure 1 and Figure 2). Mostly, there were various Ψs, and variation in the amount of Tc could be observed at Ts > To in both varieties (Figure 1 and Figure 2).

The maximum value decreased from 30 to 35 °C as water potential decreased and became more negative from 0 Mpa to −0.2 MPa. Both varieties followed the same pattern (Figure 1 and Figure 2). Further, the same reaction was perceived when the temperature at which Ψ_b(g)_ for the specific solute potential was 0 MPa, as a consequence of decreasing the germination rate at a temperature above To. It explains how the maximum temperature varies at each Ψs. Hydrothermal time models were used to determine the response of *Triticum aestivum* germination to the temperature at each solute potential (Equations (5) and (7)). We found that the model determines these interactions well with an R^2^ greater than 0.133 (Figure 1 and Figure 2 and Table 2). At all Ψs, which are restricted to an individual temperature, i.e., Tb (Figure 1a–e, and then at Ts above To, the germination rate decreases geometrically until Tc. The same pattern was followed for both varieties. An average value was 15 °C for Tb, 20 °C for To, and 35 °C for Tc in the control condition was 0 MPa for *Triticum aestivum* (Figure 1 and Table 2).

Moreover, we find a strong correlation (R^2^ > 0.133) between data at sub-TT and decreased water potential, which shares a standard set of hydrothermal time models invariable for both varieties (Figure 1 and Figure 2 and Table 2). As mentioned earlier, Ψ_b(50)_ is raised linearly at Ts > To (Figure 1 and Figure 2). Hence, Equation (8) was fitted to evaluate the tg at Ts > To. The top-quality values of the HTT model parameters are given in Table 2. According to our findings, the models could pretend well on the tg (Figure 1 and Figure 2). Nevertheless, the miserable fits of the models at 35 °C occur because, for all the data, the HTT models were fitted in both varieties to report the complete data set (Figure 1 and Figure 2).

Other germination parameters yielded similar findings. As the AAP was increased from 0 to 24, 48, and 72 h, the germination characteristics declined (Figure 3A,B and Figure 4A,B). In comparison to the control, the germination index (GI), germination rate index (GRI), mean germination time (MGT), coefficient of the velocity of germination (CVG), germination energy (GE), seed vigor index I (SVI-I), and seed vigor index II (SVI-II) fell from 0 to −0.05, −0.10, −0.15, and −0.20 MPa (Figure 3A,B and Figure 4A,B). The results of cultivar Pirsabak 15 germination characteristics are represented in Figure 3A,B) (germination index (GI), germination rate index (GRI), mean germination time (MGT), coefficient of the velocity of germination (CVG), germination energy (GE), seed vigor index I (SVI-I), and seed vigor index II (SVI-II) indicated that there were decreased when water potential declined at each temperature, and these were maximum at the optimum temperature. Similar results were found for cultivar Shankar germination parameters (Figure 4A,B).

Our findings indicate that Ts had a more significant impact on germination percentage and other germination parameters than AAP. On the other hand, farmers will benefit from using high-vigor *Triticum aestivum* L. seeds rather than low-vigor seeds, especially in DS conditions, because low-vigor seeds can drastically affect germination percentage and other germination parameters under stressful conditions.

## 4. Discussion

The optimal geographic location for a species is determined by an assessment of its germination patterns under different environmental conditions.

Mathematical models (TT, HT, and HTT) are quite effective in quantifying the effects of abiotic stress on seed germination. A knowledge of *Triticum aestivum* germination prediction using different germination models is also useful in agronomic management programs. This could help to determine and specify the impression factors of the environment on *Triticum aestivum* germination, especially the fluctuation that occurs in tg among a single seed in a seed lot [28]. Nevertheless, the thermal time model was used in many studies to report the tg at sub-optimal TT. However, at supra-optimal TT, there was a decline in these models. Furthermore, thermal time models were not predicting the reduction in germination rate when Ts exceeded the optimal temperature (Bradford 2002). Accordingly, the θH and hydrothermal time models were developed by Bradford and Still [32] and Gummerson [16] to remove the limitations and solve the related problems. Moreover, Bradford [28] reported that hydrothermal time models were accurate methods for understanding how Ts and Ψs (environmental factors) in a seed lot interact during seed germination.

Our study investigated the effect of temperature and solute potential on the germination of two winter wheat varieties for the purpose of determining their cardinal Ts, using a hydrothermal time model (Equations (5) and (7)), also known as a seed population model. Consequently, the evaluated parameters by hydrothermal time models may be incorporated into a seed germination prediction model in field conditions. Our findings show that, at each temperature level, a decline in water potential results in a lower germination rate and germination percentage in *Triticum aestivum*. This could be attributed to the fact that the seeds were being dried from a fully hydrated form, which was then unable to complete its germination process [28]. Analogous results were described in potatoes [17], zucchini [27], and watermelon [7].

In addition, we observed that the percentage of germinated seeds decreased significantly with a longer and a lower (more negative) accelerated aging period AAP. For instance, longer AAP (from 0 to 24, 48, and 72 h) decreases GP compared to the control (averaged across all levels) [33]. Further, when GP was increased from zero to −0.05, −0.1, −0.15, and −0.20 MPa, it was reduced in contrast to the control (averaged for all levels of AAP). Previous studies showed that a longer accelerated aging period (AAP) lowers the values of GP and GR in diverse crops [23,34].

Moreover, the sum germination rate declined, and the hydrotime rose largely with the declined temperature, especially at sub-TT. Because, at Ts<to< span=““>, the model measured constant Ψ</To < ψ_b(50)_ (−0.12 MPa, averaged for both varieties), which was the fundamental cause of the decline in germination rate. Later on, Ts > To, the θH was constant (2.4 MPah^−1^ in Pirsabak 15 and 0.96 MPah^−1^ in Shahkar), and only Ψ_b(50)_ was raised with the rising temperature, which resulted in an increase in germination times and a decline in germination rate. Likewise, previous studies on potatoes [17] and watermelon [7] observed an increase in θH numerical quantity at sub-optimal TT. The θH value can be used in a seed lot as a seed quality indicator, as reported previously [35]. At low temperatures, the large seeds can germinate sooner than the small seeds. On the contrary, the large seeds required a lower value of θH than the small seeds, especially at low temperatures [36].

The amount of Ψ_b(50)_ was at the minimum at Ts ≤ To (−0.12 MPa, averaged for both varieties) and then increased geometrically at supra-optimal TT, due to the thermoinhibition of *Triticum aestivum* seed germination. Other findings describe that the Ψ_b(g)_ was minimum at optimum temperature and raised linearly at supra TT, such as in potato [17], tomato [35], both onion and carrot [24], zucchini [27], and chick pea [7]. The above-described phenomenon has similar effects as declining water potential; hence, the germination rate decreased at Ts > To. If the variation between water potential and Ψ_b(50)_ is mostly small, then *Triticum aestivum* germination will be dilatory, and the germination period will be longer [28]. Additionally, according to Kebreab and Murdoch (1999), Ψs higher than Ψ_b(50)_ are evaluated in raised inactivity of the enzyme, water absorption, and accelerates the emergence of radicals. The σψ_b_ varied from 0.044 to 0.161 MPa in both varieties. In spite of this, using the estimated parameters (e.g., θH, Ψ_b(50)_ and σψ_b_) for any of the water potentials at each temperature, we can assume that the germination time courses of *Triticum aestivum*.

Moreover, we estimated the amount of Tb at 15 °C and found that *Triticum aestivum* Tb ranges from 10 to below. The To value (20 °C) was also measured to be closer to that described by [37,38], who reported the To for *Triticum aestivum* germination ranged from 20–25 °C. The Tc value at 35 °C observed was equal to the Tc value in this study exported by [38] (above 31 °C), [37] (above 30 °C), and [39] (35–42 °C). [28] proposed that using the hydrothermal time models (Equations (5) and (7)) along with a standardization factor ([1–(Ψ/Ψ_b(g))]_ tg), all tg at constant temperature and solute potential are measured on a thermal time scale, which bears the same finding in watermelon [7] and potato [17]. In the present study, we used this factor. The result showed that the elements robustly reported the time germination course of *Triticum aestivum*. Thermal time scales indicated a poor fit at 35 °C due to the model’s fitting to the entire data set to provide a more accurate statement for all levels of temperature and solute potentials. The same finding was evaluated by Rowse et al. [24], who fitted hydrothermal time models to report the germination of carrots and onions.

## 5. Conclusions

Conclusively, germination rate, percentage and other parameters (GI, GRI, CVG, SVI-I, SVI-II, GE, and MGT) are significantly influenced by water potential, temperature, and their interactions. At all Ψs and Ts, the hydrothermal time models could well report the wheat germination response. Depending on the confidence intervals of the model’s parameter, between varieties, there was no significant difference, so an average value of 40.8 MPa ℃ h^−1^ for θHTT, −0.12 MPa for Ψ_b(50)_, 0.1041 MPa ℃ h^−1^ for k_T_, 15 °C for Tb, 20 °C for To, and 35 °C for Tc was estimated for this plant. In this regard, the hydrothermal time model (HTT) provides insight into the interactive effects of T and Ψ on the germination of wheat seeds. However, the parameters of the model, on the other hand, need to be examined and compared concerning the physiological state of wheat seed populations under various environmental stress factors to predict future germination time courses in the changing climatic conditions. In light of future climate change and rising food demands, such studies may be useful to determine the optimum water potential and temperature for effective crop species development and productivity. However, the parameters of the model should be designed to assess the physio-biochemical and molecular response of the test species seed populations in relation to abiotic factors for predicting germination times in the future.

## Figures and Tables

**Table 1:** Estimated parameter values using the hydrotime (θH, Equation (3)) model to describe *Triticum aestivum* L. seed germination under different Ts and Ψs. R^2^ is the coefficient determination. SE is the standard error. σψ_b_ is the standard error. Ψ_b(50)_ is base water potential at the 50 percentile. θH is hydrotime.

Cultivar	T (°C)	θH (MPah^−1^)	Ψ_b(50)_ (MPa)	σψ_b_ (MPa)	R^2^	SE
Pirsabak 15	15 °C	6.24	−0.11	0.161	0.997	0.0089
	20 °C	8.61	−0.12	0.148	0.856	0.0600
	25 °C	6.72	−0.04	0.077	0.862	0.0588
	30 °C	2.4	−0.015	0.031	0.510	0.1167
	35 °C	2.4	−0.03	0.063	0.775	0.0750
Shahkar 13	15 °C	7.68	−0.10	0.154	0.853	0.0606
	20 °C	6.72	−0.12	0.148	0.921	0.0444
	25 °C	8.16	−0.06	0.1	0.542	0.1070
	30 °C	0.96	−0.02	0.063	0.800	0.0707
	35 °C	0.96	−0.03	0.044	0.133	0.1040

**Table 2:** Estimated parameter values using the hydrothermal time model (HTT) for describing seed germination of sesame at five-constant Ts (15, 20, 25, 30, 35 ℃) at each of the following five different Ψs (0, −0.05, −0.1, −0.15, and −0.2 MPa). R^2^ is the coefficient determination. σψ_b_ is the standard error. Ψ_b(50)_ is base water potential at the 50 percentile. θHTT is hydrothermal time.

Cultivar	Pirsabak 15	Shahkar 13	Averaged Values
HTT parameter			
Ψ_b(50)_ (MPa)	−0.120	−0.120	−0.120
σψ_b_ (MPa)	0.148	0.148	0.148
θ_HTT_ (MPa °Ch^−1^)	43.20	38.40	40.80
k_T_ (MPa °Ch^−1^)	0.104	0.1041	0.104
Cardinal Temperature			
Tb (°C)	15	15	15
To (°C)	20	20	20
Tc (°C)	35	35	35
R^2^	0.86	0.97	0.91

## Data Availability

Not applicable.
